# Green and Sustainable Separation of Natural Products from Agro-Industrial Waste: Challenges, Potentialities, and Perspectives on Emerging Approaches

**DOI:** 10.1007/s41061-017-0182-z

**Published:** 2018-01-17

**Authors:** Vânia G. Zuin, Luize Z. Ramin

**Affiliations:** 1 Department of Chemistry, Federal University of São Carlos, Rod. Washington Luís, km 235, São Carlos, 13565-905 Brazil; 2 Green Chemistry Centre of Excellence, University of York, North Yorkshire, YO10 5DD UK

**Keywords:** Green and sustainable extraction, Sustainable separation, Green analytical techniques, Biomass waste, Biorefinery, Bioeconomy and circular economy

## Abstract

New generations of biorefinery combine innovative biomass waste resources from different origins, chemical extraction and/or synthesis of biomaterials, biofuels, and bioenergy via green and sustainable processes. From the very beginning, identifying and evaluating all potentially high value-added chemicals that could be removed from available renewable feedstocks requires robust, efficient, selective, reproducible, and benign analytical approaches. With this in mind, green and sustainable separation of natural products from agro-industrial waste is clearly attractive considering both socio-environmental and economic aspects. In this paper, the concepts of green and sustainable separation of natural products will be discussed, highlighting the main studies conducted on this topic over the last 10 years. The principal analytical techniques (such as solvent, microwave, ultrasound, and supercritical treatments), by-products (e.g., citrus, coffee, corn, and sugarcane waste) and target compounds (polyphenols, proteins, essential oils, etc.) will be presented, including the emerging green and sustainable separation approaches towards bioeconomy and circular economy contexts.

## Introduction

**Fig. 1:**
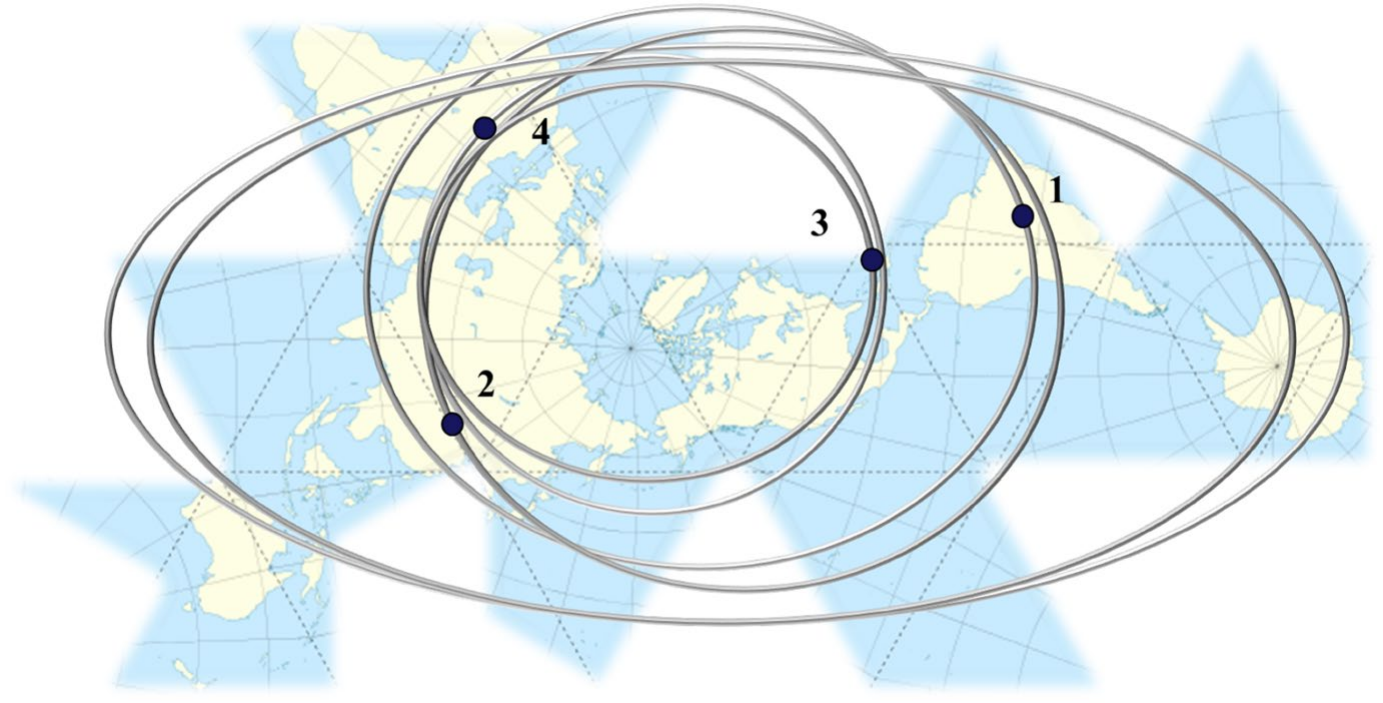
Representation of an integrated planetary flow system based on the Dymaxion map, emphasizing some coupled cycles related to food production and socio-environmental impacts among (1) Brazil, (2) China, (3) the Caribbean, and (4) the Sahara Desert. Adapted from [1]

Currently, it can be observed that global sustainability challenges are all closely interconnected, such as pollution, climate change, biodiversity loss, poverty, energy, and food security. As stated by Liu et al. [1], only holistic and disruptive approaches integrating various components of human and natural systems are effective in identifying and proposing suitable solutions for these challenges, especially those related to research, development, and innovation (RD&I) in interdisciplinary and transdisciplinary studies. To exemplify this systemic view, Fig. 1 illustrates the Earth surface that, based on the “Dymaxion map” (the Fuller Projection Map), shows the planet as a continuum without splitting any continents, seas, and oceans, where cycles are integrated through flows of matter, energy, and information [1, 2]. Here, Brazil, China, the Caribbean, and Africa interact across space, time, and organizational levels in many ways. For instance, the expansion of soybean production aggravates deforestation in Brazil, but also provides food and feedstock to China. The food trade between both countries also affects other areas, including the Caribbean and Africa. Dust particles from the Sahara Desert, also increased due to unbalanced agricultural practices, can reach the Caribbean and have an impact on coral reefs and soil fertility, diminishing tourism in this region. In addition, nutrient-rich particles from Africa can reach Brazil, improving its forest productivity.

According to the Director-General of the Food and Agriculture Organization (FAO) of the United Nations [3], after years of progress, world hunger has increased since 2015. Around 60% of the world’s starving people are from countries affected by conflict and climate change, including northeast Nigeria, Somalia, South Sudan, and Yemen with 20 million people, often suffering extreme climatic events such as droughts and floods. Not surprisingly, some of the FAO’s top priorities for the next 2 years include topics such as sustainable agriculture, climate change mitigation and adaptation, water scarcity and support of subsistence rural practices, and fisheries and forestry [3, 4]. The challenges related to this demanding context can be intensified and better understood when taking into account that the world population is expected to increase by about 30% over the next 35 years, reaching more than 9.5 billion people in 2050 and 11.2 billion in 2100 [5].

As pointed out by Xia et al. [6], the global food waste of approximately 1.3 billion tons per year is shocking in this context and, although it should be avoided or minimized, it cannot be completely prevented nowadays. Primary and secondary processing generates unpreventable food supply chain waste. This can be due to a number of factors along the supply chain, differing by the commodity and country in question. In general terms, developing countries such as some African countries suffer the greatest loss during the early, upstream part of the primary processing, corresponding to 75% of food losses during production and postharvest. Various initiatives, e.g., building better infrastructure through knowledge transfer (more efficient storage and transport technologies) and improving collaboration and market opportunities in the food supply chain could have a positive role. In industrialized countries, waste occurs especially in the consumption stage, accounting for 50% of overall loss of crops in some countries of North America, Europe, and Oceania. In this case, together with educational and cultural actions, other aspects such as developing legislation to make date labels more user-friendly for consumers (sell-by, best-before, and consume-by), redesigning packaging characteristics (avoiding the “buy 1 get 2” offers) and retailer marketing strategies should be considered [7].

It is estimated that around 140 billion tons of biomass from the agricultural sector are generated every year in the world [8, 9], and a considerable part is recognized as waste and not conflicting with food availability, e.g., leaves, roots, stalks, bark, bagasse, straw residues, seeds, wood and animal residues. Using alternative strategies to avoid additional losses and produce several high value-added chemicals could minimize the volume of non-renewable materials used today (i.e., roughly 50 billion tons of fossil fuels), enough to greatly reduce greenhouse gas emissions and dependence on non-sustainable resources. Therefore, considering their available volume and practically low costs locally and globally, associated to rich function, structure and chemical heterogeneity, all agro-industrial waste should also be considered for their chemical and material potential, as well as a source of energy [10–13].

**Fig. 2:**
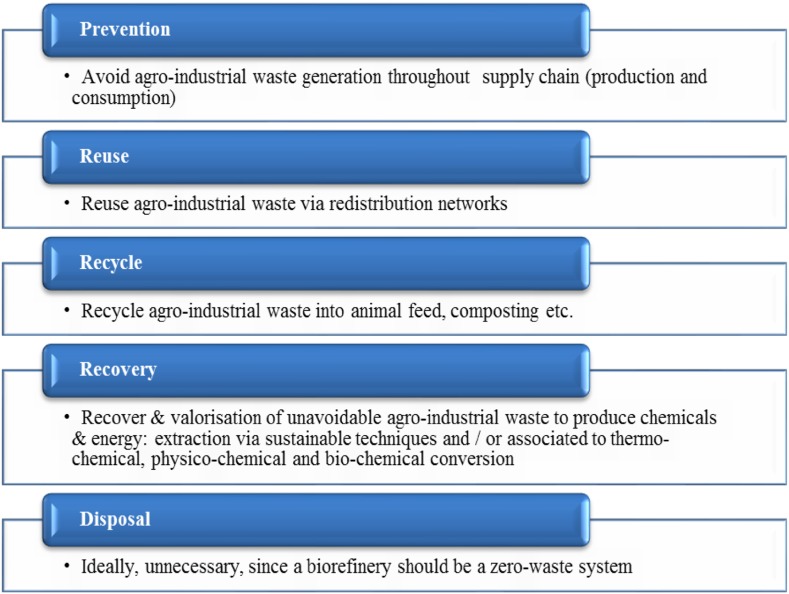
The agro-industrial waste hierarchy modified from [15]. The main idea is to promote sustainable production and consumption systems through zero-waste biorefinery

An important proposal related to waste hierarchy as a framework for residue management can be seen in Fig. 2 [14, 15], which was reformulated to include agro-industrial waste. In this case, the agro-industrial waste hierarchy has a different meaning from top to bottom, since all biomass is valued as raw material. ‘Prevention’ is an intrinsic part of optimized processes, avoiding overproduction. Therefore, the least probable option is ‘disposal’ as the supply chain is designed to attend sustainable consumption, using all bio-based material generated. Here, sustainable production also includes eco-efficiency, cleaner and green productivity, whereas sustainable consumption allows greener choices to be made by individuals based on eco-procurement, supply chain management, waste minimization, recycling, and resource efficiency measures. Both sustainable production and consumption comprises ‘life-cycle thinking’, aiming at preventing problems shifting from one life-cycle stage to another, one geographical area or environmental compartment to another.

**Fig. 3:**
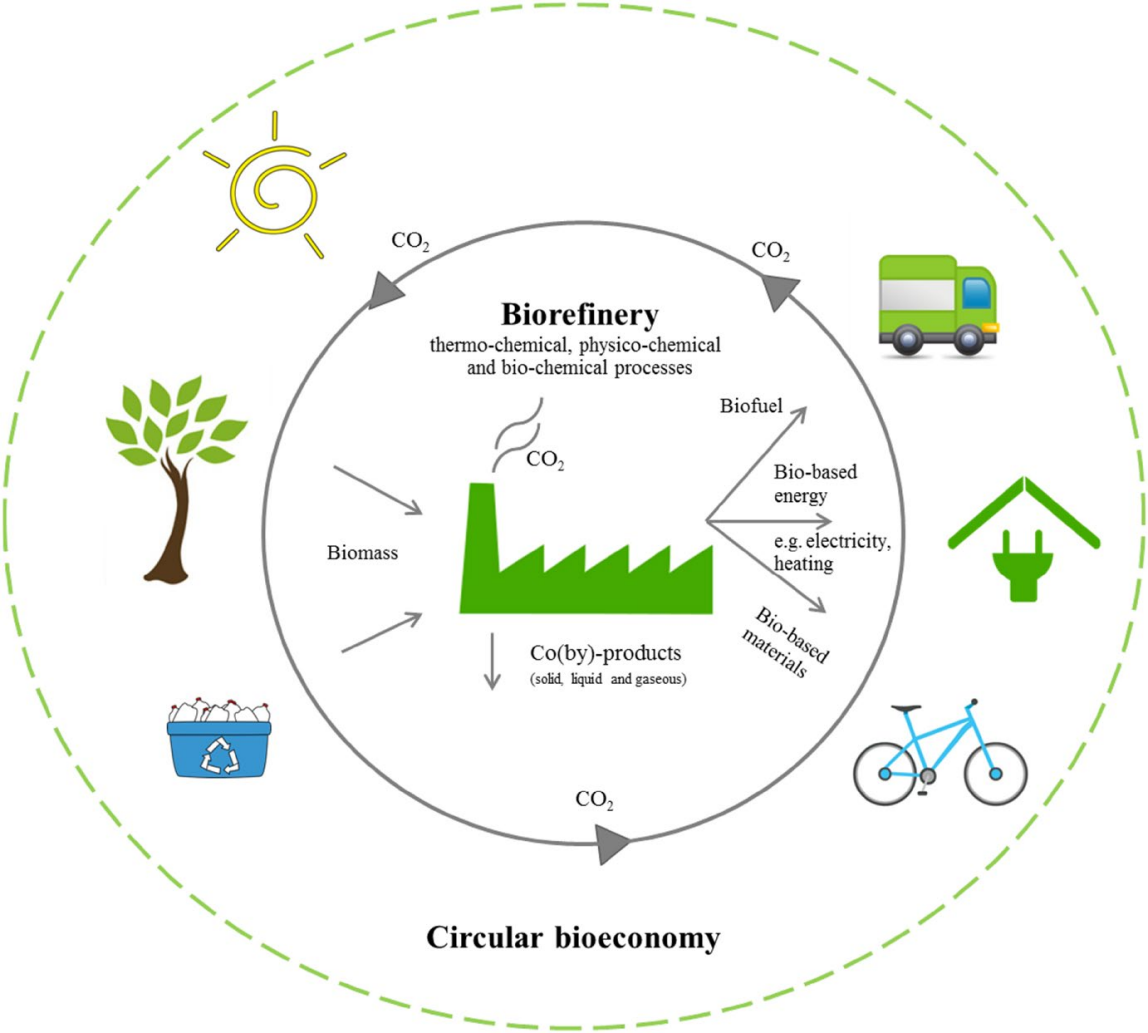
Holistic biorefinery model integrating biomass, biofuel, biomaterials and bioenergy cycle, based on green and sustainable technologies in the scope of bioeconomy and circular economy. Updated and expanded from [16, 17]

One of the most important and cited references highlighting the advances in genetics, biotechnology, process chemistry, and engineering that has helped establish a new manufacturing concept to convert renewable biomass into valuable fuels and products, known as biorefinery, was published by Ragauskas and collaborators in the mid-2000s [16]. According to these authors and other researchers [16, 17], integrating biomass and biorefinery technologies has the potential to develop sustainable bio-based energy and materials leading to a new manufacturing paradigm (Fig. 3).

In fact, this paradigm is currently connected to other strong concepts, i.e., bioeconomy and circular economy; the latter is described as an industrial system that is restorative by intention and design. This idea replaces the end-of-life notion with regeneration, focusing on the use of renewable energy, elimination of toxic chemicals, reutilization, return and eradication of “waste through the superior design of materials, products, systems, and business models” [18, 19].

As can be noted, new generations of biorefinery combine innovative biomass resources from different origins, chemical extraction and purification and/or synthesis of biomaterials, biofuels and bioenergy via benign processes. From the very beginning, the identification and quantification of all potentially high value-added compounds that could be removed from the available renewable feedstocks requires another analytical approach, also connected to green chemistry [20, 21].

## From Green to Sustainable Separation: Towards Holistic, Flexible, and Zero-Waste Biorefineries

More recently, green extraction and purification have been presented as methods based on establishing processes that reduce energy consumption, using solvents and renewable materials, as well as ensuring a safe and high-quality fraction/product [22]. The aim of their application is to obtain natural products from industrial waste, which is considered a highly attractive initiative [23].

However, a more adequate term for such extraction and purification processes towards vanguard biorefineries could be sustainable separation, adding to the previous green definition, the notion of innovation across all sectors that allows for increased value in a wide sense, enhancing human and environment benefits and providing economically accessible technologies also advantageous to industry and large scale processing systems. It includes another dimension related to the generation of more creative and healthy jobs, contributing to the construction of a positive long-term sustainability agenda, encompassing bio-circular economy, environmental and social justice [24–27].

Sustainable separation can be defined as a holistic approach grounded on the circular and flexible design and application of renewable benign materials and auxiliaries (including bio-derived solvents, solid phases, membranes) and processes [rooted on green analytical techniques and sustainability metrics and indices, e.g., life cycle analysis (LCA), chemometrics, and other interdisciplinary indicators]. The aim is to optimize the tuneable use of energy, time, reagents, devices, scale, yield and number of steps to extract, fractionate, purify or even modify the components of interest from bio-derived waste during these in situ processes, ensuring analytical reproducibility, efficiency, selectivity robustness and scalability, with online evaluation regarding measurable objectives to create safer, healthier, and more efficient products, processes, and services under fair conditions, commercially available at accessible and just prices [28–30].

Natural products are among the most attractive value-added chemicals to be considered, which can be classified as organic compounds formed by living systems divided into three main categories: (1) compounds that occur in all cells and have a central role in their metabolism and reproduction (nucleic acids, amino acids, and sugars), also known as primary metabolites; (2) high-molecular polymeric materials which form cellular structures (cellulose, lignins, and proteins) and; (3) chemicals which are characteristic of a limited number of species, called secondary metabolites [22, 30]. Many of these bioactive compounds (e.g., alkaloids, terpenoids, and phenols) have been extensively used as medicine, nutraceuticals, flavors, fragrances, cosmetics, food additives, antimicrobials, bio-pesticides, etc. However, among the biggest challenges for biomass utilization is establishing benign methods to separate, purify and modify it into chemicals, fuels, and new materials. This is partially due to, with rare exceptions, the small amounts which are lower than 0.01% of the dry weight of vegetal, associated to possible product inhibition issues, large raw material variability, feed detoxification (when necessary), instability of the target compound (or fractions) and its presence in a complex mixture [23, 30].

It is well known that the separation steps, especially extraction, correspond up to 40–80% of the total costs of most common chemical processes currently used. From the point of view of a holistic biorefinery, separation has attracted more and more attention [31]. For instance, for natural products, solvent-based extraction is one of the best options nowadays considering the nature of many bio-based chemicals and matrices, and also the fact that other separation methods, such as those based on chromatography or membranes, do not have the same advantages taking into account commercial scales [32].

It is expected that high value-added components from biomass waste such as essential oils, polyphenols, and other food or medicinal-related products are extracted first, followed by polysaccharides, lignocelluloses or waxes via advanced separation and depolymerization processes. Among them, green solvents in general, supercritical CO_2_, subcritical water, microwave (MW)-assisted acidolysis and gas-expanded liquids have been mentioned [33]. Green solvents offer important separation advantages, including near-supercritical or supercritical fluids, which have outstanding mass transport properties, polarity, and easiness of solvent removal after extracting the compound of interest [34]. Another interesting solvent is water, but the range of compounds that are soluble in this medium is quite limited. Nevertheless, the use of subcritical water has been demonstrated to be advantageous for organic modification to depolymerize, hydrolyze, gasify, and carbonize biomass to produce bioactive compounds, sugars, biogas, and other valuable solids [16, 35].

Integrating two or more green techniques combining different strategies has played an important role in overcoming the main drawbacks of a single technique towards sustainable separation. For instance, for high-pressure solvent extraction in which the extractants do not reach supercritical conditions, the temperature, time, and solvent consumed can be dramatically reduced associating ultrasound-assisted treatment [28, 36]. In fact, more attention has been paid to green extraction, purification, or modification of natural products derived from agro-industrial waste nowadays, opening up new opportunities for sustainable approaches designed for bioeconomy and circular economy models. The aim of this paper is to present an overview of the design and application of green and sustainable separation of natural products for vanguard zero-waste biorefineries. The main analytical techniques and procedures described over the last 10 years will be described in detail, showing the potentialities, challenges, and perspectives in this topical and emergent scenario.

## High Value-Added Approaches for Green and Sustainable Separation of Natural Products from Waste: What can be Observed from the Literature?

**Fig. 4:**
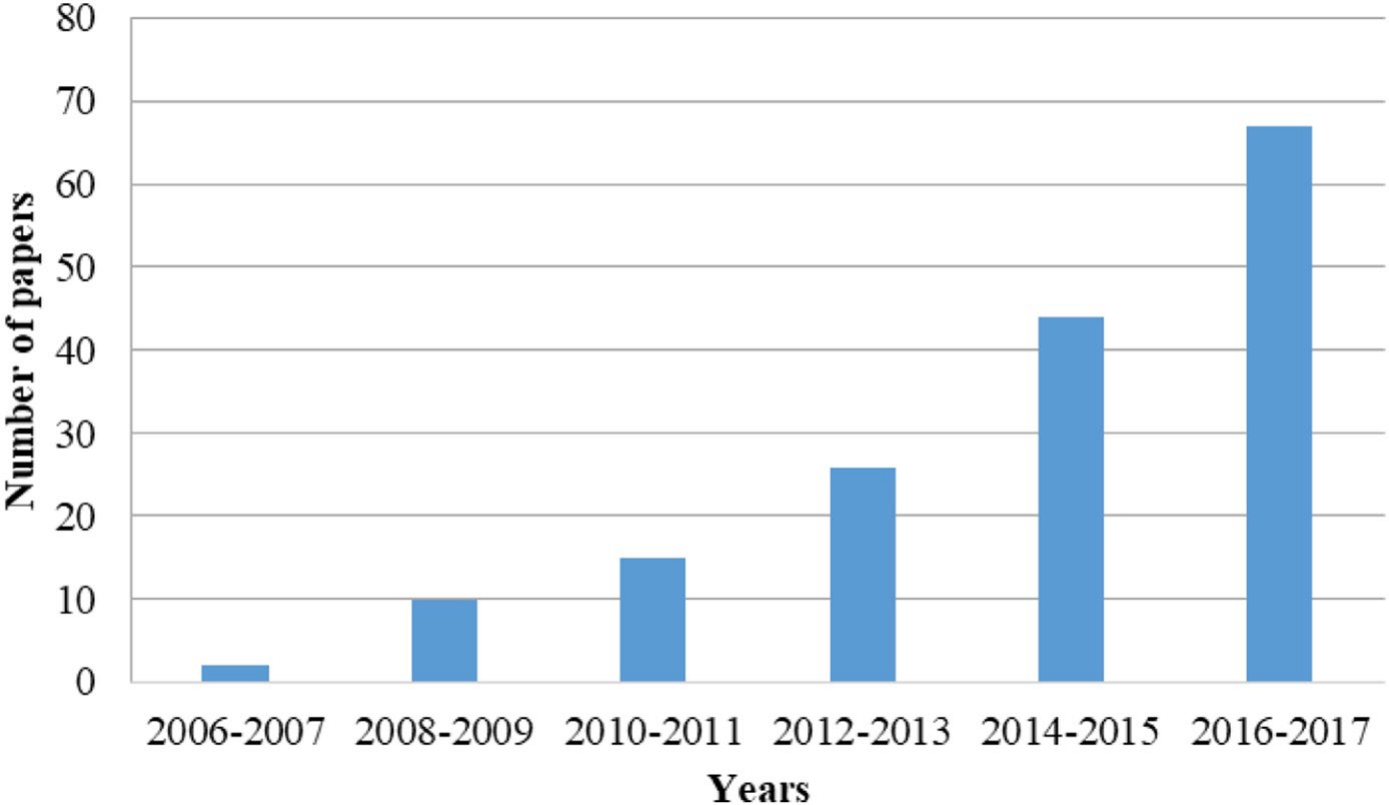
Number of publications per year focusing on green and sustainable separation (extraction, fractionation and purification) of natural products from waste (ISIS Web of Knowledge, January 2006 to December 2017)

More recently, trends in green and sustainable extraction, fractionation and purification techniques have largely focused on minimizing the use of solvents, energy and materials that are intrinsically benign to human health and the environment [37]. In order to analyze the *status quo* and perspectives related to natural product separation from waste, a systematic literature review was conducted using the ISIS Web of Knowledge platform (reviews and papers) from 2006 to 2017, combining the descriptors “natural product” and “green extraction/separation” (or “sustainable extraction/separation” or “eco-friendly extraction/separation”) and “waste” (or “residue”). Figure 4 shows the number of publications during this period. There were more than 160 research papers and reviews that, to the best of our knowledge, are reasonably representative to show the strongest tendencies in this field over the last decade. It can be clearly observed that there has been an increase in the number of manuscripts over the last 10 years, covering the principles, advances, and applications of these green methods.

The obtained data reflect the growing interest and potential of green and sustainable methods to separate natural products from waste. One tendency observed in particular was the innovative ways to remove (integrating extraction, purification and/or modification in the same integrated system) and use such compounds in more contemporary sectors, promoting human and environmental health instead of general and old-fashioned remediation [19, 38]. As a result, new applications for food, nutraceutical, and agricultural sectors have been further explored, based on their advantageous properties as natural colorants, flavors, aromas, antioxidants, antifungals, bioformulations (bio-pesticides) or simply their use as precursors to generate other compounds for similar uses. Some details related to patents, (non-) clinical trials, sustainable indicators, scaling-up, regulatory, agro-industrial variability and availability, traceability, seasonality, good laboratory and manufacturing practices, additional economical and marketing issues have also been discussed.

Table 1 presents the research papers and reviews published during this period, highlighting their main focus, the green or sustainable techniques/approaches adopted, raw materials (mostly agro-industrial waste) and target compounds studied. The most common raw materials described as chemical feedstocks were waste derived from plants, for instance, food, mainly fruits (citrus, mango, papaya, grape, passiflora, banana, tomato, olive), grains (corn, soybean, sunflower, coffee) and other abundant materials (sugarcane bagasse, tea, wood bark, rice and wheat straw). Additional issues that affect the quality of the final products were also discussed, namely the procedure used for waste collection, selection, storage, drying, matrix characteristics (particle size, shape, specific surface area and porosity). The latter aspects play an important role in extraction efficiency due to the mass and heat transfer processes. Understanding the nature of raw material is crucial to avoid negative influences impacting the quality and yield during the removal of the target compounds, e.g., caused by co-extracted contaminants or due to the presence of some components in these matrices, such as water or high molecular weight compounds [39].

**Table 1:** Research papers and reviews focusing on green and sustainable separation of natural products from agro-industrial waste published from January 2006 to December 2017 (ISIS Web of Knowledge)

Year	Crop	Waste stream	Target compounds	Geographical location	Green or sustainable separation approach	References
2017	Olives	Olive kernels	Phenolic compounds and oil	France and Spain	Aqueous liquid solid extraction (LSE), mechanical expression (ME), supercritical CO_2_ (SC-CO_2_) and gas-assisted mechanical expression (GAME)	Gas-assisted mechanical expression (GAME) for the selective recovery of lipophilic and hydrophilic compounds from olive kernel [145]
2017	Figs	Leaves	Bioactive compounds	China	Deep eutectic solvent with microwave and ultrasound extraction Time: 10 min (MW) and 60 min (US) Temperature: 40–80 °C Power: 250 W (MW) and 700 W (US)	Enhanced and green extraction polyphenols and furanocoumarins from Fig (*Ficus carica* L.) leaves using deep eutectic solvents [136]
2017	*Polygonum multiflorum*	Herbal raw materials	Stilbene glycoside and anthraquinones	China	Ionic liquids with ultrasonic extractor Time: 1–120 min Power: 40–120 W	Sequential extraction and separation using ionic liquids for stilbene glycoside and anthraquinones in *Polygonum multiflorum* [131]
2017	Several sources	Not defined	Mostly bioactive compounds	Spain	Review Critical overview about the greenness of water as extraction solvent	Water as green extraction solvent: Principles and reasons for its use [146]
2017	Pomelo	Flavedo	Essential oil	China	Microwave irradiation Power: 240–700 W Time: 24 min	A process to preserve valuable compounds and acquire essential oils from pomelo flavedo using a microwave irradiation treatment [52]
2017	*Selaginella doederleinii*	Not defined	Biflavonoids	China	Ionic liquids and microwave-assisted extraction Power: 300–700 W Time: 30–50 min Temperature: 40–60 °C	Optimization of ionic liquid-assisted extraction of biflavonoids from *Selaginella doederleinii* and evaluation of its antioxidant and antitumor activity [132]
2017	*Pogostemon cablin*	Leaves	Essential oils	Indonesia	Microwave-assisted hydrodistillation (MAHD) and solvent-free microwave extraction (SFME) Power: 600 W (MAHD) and 264 W (SFME) Time: 66 min (MAHD) and 45 min (SFME); solvent: water	Comparison of conventional and microwave-assisted distillation of essential oil from *Pogostemon cablin* leaves: analysis and modeling of heat and mass transfer [147]
2017	*Juglans regia* L.	Fresh male flowers and unripe walnut seeds	Phenolic content and water-soluble polyphenols	Italy	Microwave-assisted extraction Frequency: 2.45 GHz Max. power: 500 W Solvent: ethanol/water Temperature: 60–100 °C Time: 6–30 min	Process intensification by experimental design application to microwave-assisted extraction of phenolic compounds from *Juglans regia* L. [148]
2017	Walnuts	Walnut de-pellicle	Flavonoids	China	Macroporous resins Pretreated with 5% HCl and 5% NaOH solutions	Recovery of flavonoids from walnuts de-pellicle wastewater with macroporous resins and evaluation of antioxidant activities in vitro [149]
2017	Ginseng	Roots	Bioactive compounds	Brazil	Sequential extraction system using ethanol followed by water Temperature: 333 K Time: 5–240 min	Techno-economic evaluation of obtaining Brazilian ginseng extracts in potential production scenarios [150]
2017	Food ingredients and natural products	Not defined	Nutraceutics, cosmetic, pharmaceutical, and bioenergy applications	France	Review current knowledge on ultrasound-assisted extraction	Ultrasound-assisted extraction of food and natural products. Mechanisms, techniques, combinations, protocols and applications. A review [151]
2017	Coffee	Coffee chaff	Antioxidants	Portugal	Solid–liquid extraction and multi-frequency multimode modulated (MMM) Frequency: 19.8 kHz Power: 250 and 500 W Time: 60–600 s	Multi-frequency multimode modulated technology as a clean, fast, and sustainable process to recover antioxidants from a coffee by-product [152]
2017	Apples	Wild apple fruit dust	Bioactive compounds, polyphenolic antioxidants	Serbia	Microwave-assisted extraction Time: 15–35 min Ethanol conc.: 40–80% Irradiation power: 400–800 W	Microwave-assisted extraction of wild apple fruit dust production of polyphenol-rich extracts from filter tea factory by-products [153]
2017	Wood	Wood biomass	Lignin oligomers	China	Microwave-assisted treatment with deep eutectic solvent Solvent: choline chloride and oxalic acid dehydrate Temperature: 80 °C Power: 800 W Time: 3 min	Efficient cleavage of lignin-carbohydrate complexes and ultrafast extraction of lignin oligomers from wood biomass by microwave-assisted treatment with deep eutectic solvent [137]
2017	Wood	Oak wood from cooperage by-products	Furanic compounds, *cis*- and *trans*- B-methyl-*y*-octalactones, terpenes and norisoprenoids, benzenic compounds	Spain	Pressurized liquid extraction Solvent: water, ethanol/water (80:20) and ethyl lactate Temperature: 60–120 °C Pressure: 10.34 MPa Flush volume: 60% Purging time: 80 s	Extraction of natural flavorings with antioxidant capacity from cooperage by-products by green extraction procedure with subcritical fluids [154]
2017	*P. armeniaca, P. persica, P. domestica, Triticum aesativum*	Fruit and vegetables seeds and peels	Phenolic compounds	Pakistan	Ultrasonic water bath Solvent: 65% (v/v) ethanol (methanol and acetone) Extraction time: 30 min Temperature: 50 °C	Extraction and quantification of phenolic compounds from *Prunus armeniaca* seed and their role in biotransformation of xenobiotic compounds [71]
2017	Lignocellulose materials	Lignocellulosic biomass such as crops or forestry residues	High value-added bio-based products (e.g., bioethanol, biogas, acetic acid, acetic acid, or activated carbon)	Mexico and Pakistan	Review Focus on transformation based on syngas platform (thermochemical platform) and sugar platform (biochemical platform)	Lignocellulose: a sustainable material to produce value-added products with zero-waste approach [155]
2017	Olives	Olive by-product (paté)	Fatty acids and phenolic compounds	Spain and Italy	Soxhlet extraction (percolation with petroleum ether, under reflux)	Macro and micro functional components of a spreadable olive by-product (pate) generated by new concept of two-phase decanter [156]
2017	Tucumã palm fruit	Tucumã’s endocarp	Cellulose	Brazil and USA	Alkaline extraction (135 °C, autoclave, 2 bar, 2 min, 20% of aqueous NaOH, 1:30 straw to liquor (g/ml), 30 min)	New approach for extraction of cellulose from tucuma’s endocarp and its structural characterization [115]
2017	Grapes	Seeds	Resveratrol	China	Subcritical water extraction Pressure: 0.5–1.5 MPa Time: 20–30 min Temperature: 130–170 °C	Optimization of subcritical water extraction of resveratrol from grape seeds by response surface methodology [100]
2017	Mango, rambutan, santol	Peels	Antioxidant activity	Thailand	Solid–liquid extraction Ethanol (95%)	Study effect of natural extracts on the antioxidant activity in pork balls [157]
2017	Tomatoes	Pericarps without seeds	Nutrient-rich antioxidant ingredients	Portugal, Spain, Ireland	Microwave extraction (600 rpm, 200 W) Time: 0–20 min Temperature: 60–180 °C Ethanol conc.: 0–100% Solid/liquid ratio: 5–45 g/l	Valorization of tomato wastes for development of nutrient-rich antioxidant ingredients: a sustainable approach towards the needs of today’s society [158]
2017	*Citrus latifolia*, *Rubus* sp., *Origanum vulgare* and *Heterotheca inuloides*	Peel and broken down vegetable material	Fatty acids and antioxidants compounds	Mexico, Belgium	SC-CO_2_ Extraction time: 1 h Flow: 25 g/min Pressure: 10–40 MPa Temperature: 35–60 °C Co-sol.: 0–8 g/min Percent flow: 0–32%	Thermodynamics and statistical correlation between supercritical CO_2_ fluid extraction and bioactivity profile of locally available Mexican plant extracts [159]
2017	Pomegranates	Peels	Carotenoids	Greece	Ultrasound-assisted extraction (139 W, 20 kHz); solvents: vegetable oils Extraction time: 10–60 min Temperature: 20–60 °C	Green ultrasound-assisted extraction of carotenoids from pomegranate wastes using vegetable oils [72]
2017	Pomegranates	Both edible and non-edible parts	Polyphenols	Greece	Semi-automatic extractor Solvents: H_2_O, β-CD, HP-β-CD Extraction time: 363 min Temperature: 25 °C	Green extraction of polyphenols from whole pomegranate fruit using cyclodextrins [121]
2016	Quince	Leaves	Natural dyes and bioactive compounds	Romania	Aqueous extraction Extraction time: 60–240 min Temperature: 4–100 °C	Dyeing and antibacterial properties of aqueous extracts from quince (*Cydonia oblonga*) leaves [160]
2016	Corn	Steep liquor	Vanillic acid, *p*-coumaric acid, ferulic acid, sinapic acid and quercetin	Spain, Portugal, and Italy	Liquid–liquid extraction Solvents: chloroform (56 °C, 60 min) Ethyl acetate (25 °C, 45 min)	A multifunctional extract from corn steep liquor: antioxidant and surfactant activities [161]
2016	Palm	Oil palm empty fruit bunches	Cellulose with polypropylene as biocomposite material	Malaysia, Pakistan	Ultrasonic treatment (40 kHz) solvent: hydrogen peroxide Extraction time: 1–3 h Room temperature	Autoclave and ultra-sonication treatments of oil palm empty fruit bunch fibers for cellulose extraction and its polypropylene composite properties [73]
2016	Tomatoes	Seeds and peels	Carotenoids/proteins	Tunisia and Germany	Supercritical CO_2_ extraction 80 °C, 400 bar, 4 g CO_2_/min for 2 h	Biorefinery cascade processing for creating added value on tomato industrial by-products from Tunisia [82]
2016	Black tea	Black tea processing waste	Antioxidant and antimicrobial phenolic compounds	Turkey and USA	Solvent extraction Solvents: H_2_O, ethanol Extraction time: 2 h Temperature: 70 °C	Black tea processing waste as a source of antioxidant and antimicrobial phenolic compounds [46]
2016	Rapeseed	Rapeseed oil cakes	Protein- and lignin-rich fractions	France	Ultrafine miffing and electrostatic separation Solvents: NaOH, diethylether, hexane Extraction time: 5 h Temperature: 60 °C	Chemical- and solvent-free mechanophysical fractionation of biomass induced by tribo-electrostatic charging: separation of proteins and lignin [139]
2016	Sunflower	Seeds	Sunflower protein-based ingredients	USA	Review Green pigmentation associated with the interaction of sunflower protein and oxidized chlorogenic acid (CGA) by outlining the sunflower oil and protein meal market, CGA reactions contributing to greening, methods for CGA extraction, and the effect of processing on sunflower protein quality and the greening reaction	Chlorogenic acid oxidation and its reaction with sunflower proteins to form green-colored complexes [162]
2016	Passion fruit	Peels	Pectin	Malaysia	Acidic and enzymatic extraction Citric solution, celluclast Extraction time: 30–120 min Temperature: 35–85 °C	Comparison of acidic and enzymatic pectin extraction from passion fruit peels and its gel properties [107]
2016	Red grape	Pomace	Polyphenols and anthocyanin pigments	Greece	Ultrasound-assisted extraction (140 W, 37 kHz) Solvent: aqueous glycerol Extraction time: 60 min Temperature: 45 °C	Development of a green process for the preparation of antioxidant and pigment-enriched extracts from winery solid wastes using response surface methodology and kinetics [74]
2016	Orange and lemon	Fresh and waste peel	Pectin and d-limonene	Portugal and Italy	Microwave Solvent: water Extraction time: 1 h Temperature: 80 °C	Eco-friendly extraction of pectin and essential oils from orange and lemon peels [53]
2016	Coffee	Spent coffee grounds	Oil	China	Ultrasonication extraction Solvent: hexane Extraction time: 15–75 min	Effect of oil extraction on properties of spent coffee grounds-plastic composites [98]
2016	Tomato	Waste of tomato paste plants	Lycopene	Iran and Canada	Microemulsion technique (MET) Solvents: water, saponin: glycerol, surfactant: lycopene Extraction time: 30 min Temperature: 25 °C	Enhanced lycopene extraction from tomato industrial waste using microemulsion technique: optimization of enzymatic and ultrasound pre-treatments [163]
2016	Red capsicum (*Capsicum annuum*)	Processing residue	Carotenoids	India	Enzymatic liquefaction Pectinase, viscozyme L, cellulose extraction Time: 1 h Temperature: 60 °C	Enzyme-assisted extraction of carotenoid-rich extract from red capsicum (*Capsicum annuum*) [108]
2016	Rice	Husk	Cellulose	India	Eco-friendly method montmorillonite, LiOH, H_2_O_2_ Extraction time: 6 h Temperature: 80 °C	Extraction of cellulose from agricultural waste using montmorillonite K-10/LiOH and its conversion to renewable energy: biofuel by using *Myrothecium gramineum* [122]
2016	Tea (yarrow and rose hip)	By-products from filter-tea factory	Chlorophylls and carotenoids	Serbia	Supercritical fluid extraction Extraction time: 5 h Temperature: 40 and 60 °C Pressure: 100–300 bar CO_2_ flow rate: 0.194 hk/h	Extraction of minor compounds (chlorophylls and carotenoids) from yarrow-rose hip mixtures by traditional versus green technique [83]
2016	Corn, sugarcane, sorghum, pearl millet, green gram, groundnut sesame	Bagasse, stover, stalk and shell	*Para*-coumaric acid (pCA)	India and USA	Alkaline hydrolysis pH 3, alkali conc.: 0.5–4 M Hydrolysis duration: 4–24 h Sugaring-out for separation of pCA from hydrolysate	Extraction of *p*-coumaric acid from agricultural residues and separation using ‘sugaring out’ [116]
2016	Winery	Grape wastes and by-products	Antioxidant compounds and polyphenols	Denmark, China, France and Brazil	Review Conventional (solid liquid extraction, heating, grinding, etc.) and non-conventional (pulsed electric fields, high voltage electrical discharges, pulsed ohmic heating, ultrasounds, microwave-assisted extractions, sub- and supercritical fluid extractions, as well as pressurized liquid extraction) methods	Green alternative methods for the extraction of antioxidant bioactive compounds from winery wastes and by-products: a review [164]
2016	1st to 3rd generation biodiesel feedstocks	Mostly microalgae	Biodiesel	Malaysia and Japan	Review Integration of enzymatic reactors with supercritical fluid technology	Green biodiesel production: a review on feedstock, catalyst, monolithic reactor, and supercritical fluid technology [84]
2016	*Jatropha curcas*, oil palm	Seeds, empty fruit bunch	Bio-oil	Malaysia	Microwave extraction Solvent: water Extraction time: 60–140 min Power: 200–700 W	Green bio-oil extraction for oil crops [54]
2016	Green tea	Green tea residue	Protein	The Netherlands	Alkaline protein extraction Solvent: NaOH Extraction time: 2 h Temperature: 95 °C	Improving yield and composition of protein concentrates from green tea residue in an agri-food supply chain: effect of pre-treatment [117]
2016	Eucalyptus wood	Eucalyptus chips	Hemicelluloses	Uruguay	Green liquor extraction Solvents: water and green liquor (Na_2_CO_3_, Na_2_S, and NaOH) extraction time: 30–150 min temperature: 100–160 °C	Integrated forest biorefineries: green liquor extraction in eucalyptus wood prior to kraft pulping [123]
2016	Watermelons	Juice	Lycopene	Brazil	Microfiltration, diafiltration, reverse osmosis α-Al_2_O_3_ membranes T1-70 (35 °C) Polyamide composite membranes (35 °C, 60 bar)	Integrated membrane separation processes aiming to concentrate and purify lycopene from watermelon juice [140]
2016	Larch wood	Sapwood, heartwood, bark and branches	Phenolic compounds	Slovenia	Pressurized hot water Extraction time: 30 min Temperature: 100 °C	Isolation of phenolic compounds from larch wood waste using pressurized hot water: extraction, analysis and economic evaluation [165]
2016	Tomatoes	Pomace	Lycopene	Iran	Microemulsion technique H_2_O and surfactants Extraction time: 30 min Temperature: 35 °C	Microemulsion-based lycopene extraction: effect of surfactants, co-surfactants, and pretreatments [166]
2016	Melons	Rind	Carbohydrates, phenolic compounds, and fatty acids	Spain	Solvent extraction Solvent: cyclohexane, ethanol Extraction time: 2 h Microwave radiation: 190 °C, 20 min, 200 W	Microwave heating for the catalytic conversion of melon rind waste into biofuel precursors [167]
2016	Tomatoes, fungus *Blakeslea trispora*	Processing waste	Lycopene	Greece	Review Emphasis on final product safety and ecofriendly processing (solvent extraction, SFE, MAE, high-pressure processing, ultrasound, electrical methods)	Natural origin lycopene and its “green” downstream processing [168]
2016	Oranges	Peel	Pectin	Italy	Conventional hydrodistillation, MAE, US Solvents: water Extraction time: 5–155 min Temperature: 90–333 °C	Novel configurations for a citrus waste based biorefinery: from solventless to simultaneous ultrasound and microwave-assisted extraction [55]
2016	Lemons, olives, onion, red grape, coffee, and wheat	Peel, leaves, solid wastes, pomace, spent filter and bran	Polyphenolic compounds	Greece	Ultrasound extraction (140 W, 37 kHz) eutectic mixtures Extraction time: 90 min Temperature: 80 °C	Novel glycerol-based natural eutectic mixtures and their efficiency in the ultrasound-assisted extraction of antioxidant polyphenols from agri-food waste biomass [75]
2016	Potatoes	Peels	Polyphenolic antioxidants	Greece	Ultrasound extraction (140 W, 37 kHz) Solvents: ethanol and glycerol Extraction time: 90 min Extraction temperature: 50–80 °C	Optimization of a green ultrasound-assisted extraction process for potato peel (*Solanum tuberosum*) polyphenols using bio-solvents and response surface methodology [76]
2016	Grapes	Seeds	Grape seed oil	Croatia	Supercritical CO_2_ Extraction time: 90 min Temperature: 35–64 °C Pressure: 158–441 bar CO_2_ flow rate: 1.94 kg/h	Optimization of supercritical CO_2_ extraction of grape seed oil using response surface methodology [85]
2016	*Crocus sativus*	Petals (underutilized bulk agro-waste)	Phenolic compounds	Iran	Subcritical water extraction Extraction time: 20–60 min Temperature: 120–160 °C	Optimization of the subcritical water extraction of phenolic antioxidants from *Crocus sativus* petals of saffron industry residues: Box–Behnken design and principal component analysis [101]
2016	Bananas	Peels	Antioxidants	Malaysia and Turkey	Solvent extraction Solvents: acetone, ethanol, hexane, methanol, H_2_O Extraction time: 1–5 h	Optimization of extraction parameters on the antioxidant properties of banana waste [47]
2016	Pea vine	Pea vine waste	Potential platform molecules (5-hydroxy furfural; ethanoic acid); sugars (levoglucosenone, rhamnose, xylose, fructose); biopolymer with pectinaceous and starch-like characteristics	United Kingdom	Pseudo-subcritical water extraction Temperature: 125–175 °C Pressure: 20–60 bar Flow rate: 1–5 ml/min	Potential utilization of unavoidable food supply chain wastes-valorization of pea vine wastes [6]
2016	Keratin-containing products stored in large waste deposits	Processing waste	Keratin	Romania	Review Keratins solubilization (protected and unprotected methods) followed by dehydro-thermal, physical-type bonding or chemical treatments	Practical ways of extracting keratin from keratinous wastes and by-products: a review [169]
2016	*Taxus baccata* L.	Case study based on European yew	10-deacetylbaccatin III (10-DAB)	Germany	Review Theoretical approach in thermodynamics and process modelling as an alternative process design	Process design for integration of extraction, purification and formulation with alternative solvent concepts [170]
2016	Olives	Olive mill waste water	Biophenols (hydroxytyrosol and tyrosol)	Italy	Liquid–liquid extraction Solvents: *n*-hexane, EtOAc	Quick assessment of the economic value of olive mill waste water [171]
2016	Olives	Olive mill waste water	Tyrosol	Spain, United Kingdom and Spain	Hydrophobic ionic liquids Solvents: ILs Extraction time: 2 h Temperature: 303–323 K	Recovery of tyrosol from aqueous streams using hydrophobic ionic liquids: a first step towards developing sustainable processes for olive mill wastewater (OMW) management [133]
2016	Cupuassu	Seeds	Cupuassu butter (phenolic content/tocopherols/fatty acids)	Brazil	Supercritical CO_2_ extraction Temperature: 50 and 70 °C Pressures: 20–40 MPa	Supercritical CO_2_ extraction of cupuassu butter from defatted seed residue: experimental data, mathematical modeling and cost of manufacturing [86]
2016	Coffee	Spent coffee grounds	Oil fraction	Portugal, Brazil, Portugal	Supercritical CO_2_ Extraction time: 1 h Temperature: 55 °C Pressure: 250 bar Flow rate: 15 kg/h	The green generation of sunscreens: using coffee industrial sub-products [87]
2016	Ginger	Not defined	Essential oil, phenolics, fibers and phenolic acids	France	Microwave hydrodiffusion and gravity processing (MHG) and UAE Solvents: water Extraction time: 83 and 90 min Temperature: up to 100 and 50 °C	Towards a “dry” bio-refinery without solvents or added water using microwaves and ultrasound for total valorization of fruit and vegetable by-products [56]
2016	Passion fruit	Passion fruit seeds and passion fruit seed cake (the residue from the seed oil production by cold pressing)	Oil and extract with promising antioxidant and antimicrobial activities	Brazil and USA	SFE, LPE, MAC, UE Solvents: sCO_2_, hexane, ethyl acetate, ethanol, H_2_O Extraction time: 45 min–7 days temperature: room temp.− 50 °C	Valorization of passion fruit (*Passiflora edulis* sp.) by-products: sustainable recovery and biological activities [88]
2016	Wood	Broken pallets, crates, and waste timber from building and demolition works	Renewable energy source	Romania	Review Overview of the technical and economic opportunity of using wood waste as a renewable energy source	Wood waste as a renewable source of energy [172]
2015	Plants of spontaneous flora, cultivated plant, and wastes resulted in agricultural and food industry	General bio-derived materials	Polyphenols	Romania	Review Microwave-assisted extraction (MAE), supercritical fluid extraction (SFE), and ultrasound-assisted extraction (UAE)	A comparative analysis of the ‘green’ techniques applied for polyphenols extraction from bioresources [173]
2015	Onion	Onion solid wastes	Polyphenol- and pigment-enriched extracts with antioxidant activity	Greece	Ultrasound extraction (140 W, 37 kHz) Extraction time: 60 min Temperature: 45 °C	A green ultrasound-assisted extraction process for the recovery of antioxidant polyphenols and pigments from onion solid wastes using Box–Behnken experimental design and kinetics [174]
2015	Six types of plant fibers (bast, leaf, seed, straw, grass, and wood) and animal fibers and regenerated cellulose fibers	Seed (coir) and animals (chicken feather) as they are secondary or made from waste products	Fibers	Sweden	Review Dew, stand, cold and warm water, steam, enzyme, mechanical, ultrasound chemical and Surfactant retting	A review of natural fibers used in biocomposites: plant, animal and regenerated cellulose fibers [175]
2015	Non edible vegetables	Seeds	Biodiesel	Egypt	Review	A review on green trend for oil extraction using subcritical water technology and biodiesel production [102]
2015	Neem	Neem seed cake (NSC)	Neem Protein (NP)	USA	Alkaline extraction Solvents: H_2_O and NaOH Extraction time: 60 min Temperature: 75 °C	Bio-based polymeric resin from agricultural waste, neem (*Azadirachta indica*) seed cake, for green composites [118]
2015	Oranges	Peel	Essential oil, polyphenols and pectin	Algeria and France	MHG, UAE, MAE Solvents: “in situ” water Extraction time: 25 and 3 min Temperature: 59 °C	Bio-refinery of orange peels waste: a new concept based on integrated green and solvent free extraction processes using ultrasound and microwave techniques to obtain essential oil, polyphenols and pectin [57]
2015	Corn, sugarcane, sorghum, soybean, rice, barley, potato, other lignocellulose, vegetable oils, oilseed	By-products (bagasse, straw, cobs, stalks, stover, grass etc.)	Biofuel, 1,3-propanediol, succinic acid, adhesives, solvents, surfactants, ethyl lactate, erucic acid, amylose ethers, among others	Denmark	Review Focus on integrating sustainability assessment procedures and tools (LCA and evaluation approaches)	Biorefining in the prevailing energy and materials crisis: a review of sustainable pathways for biorefinery value chains and sustainability assessment methodologies [144]
2015	Agro-industrial products	Agro-industrial co-products	Phenolic compounds	Brazil	Solid-state fermentation, even as friendly enzyme-assisted extractions	Biotransformation and bioconversion of phenolic compounds obtainment: an overview [176]
2015	Cashew-nut	Husk	Natural dyes	India	Enzyme-assisted extraction cellulase and pectinase Solvent: water Extraction time: 60–180 min pH 9.5	Cashew-nut husk natural dye extraction using Taguchi optimization: green chemistry approach [109]
2015	Beet	Sugar beet pulp	Monosaccharides present in hydrolyzed SBP pectin: l-rhamnose, l-arabinose, d-galactose and d-galacturonic acid	United Kingdom	Centrifugal partition chromatography ascending mode, 1000 rpm Mobile phase flow rate: 8 ml/min	Centrifugal partition chromatography in a biorefinery context: separation of monosaccharides from hydrolyzed sugar beet pulp [141]
2015	Mangoes (*Mangifera indica* L.) and rye grains (*Secale cereals* L.)	Peels and grains	Alk(en)ylresorcinols (ARs)	Germany	Ultrasound-assisted extraction Solvent: dichloromethane Extraction time: 15 s cooled in ice bath	Development and validation of an HPLC method for the determination of alk(en)ylresorcinols using rapid ultrasound-assisted extraction of mango peels and rye grains [78]
2015	Olives	Waste from olive oil production	High-added value compounds (polyphenols, fatty acids, coloring pigments (chlorophylls and carotenoids), tocopherols, phytosterols, squalene, volatile and aromatic compounds)	Spain, France, Morocco and Portugal	Review Conventional (solvent, heat, grinding) and non-conventional methodologies (ultrasounds, microwaves, sub- and supercritical fluid extractions, pressurized liquid extraction, pulsed electric fields and high voltage electrical discharges)	Emerging opportunities for the effective valorization of wastes and by-products generated during olive oil production process: non-conventional methods for the recovery of high-added value compounds [142]
2015	Asparagus	Dried segments (residues)	Antioxidant compounds	China	Solid–liquid extraction Solvents: acetone, methanol or ethanol Extraction time: 2 h Temperature: 70 °C	Extraction and analysis of antioxidant compounds from the residues of *Asparagus officinalis* L. [177]
2015	Grapes	Skin	Anthocyanins	Korea	Deep eutectic solvents (DESs) Extraction time: 45 min room temperature	Highly efficient extraction of anthocyanins from grape skin using deep eutectic solvents as green and tunable media [138]
2015	Green tea	Green tea leaf residue	HG pectin, RGII pectin, organic acids, cellulose and hemi-cellulose	The Netherlands	Alkaline extraction Solvents: 0.1 M NaOH Extraction time: 2 h (protein), 5 min–24 h (carbohydrates or lignin) Temperature: 95 °C	How does alkali aid protein extraction in green tea leaf residue: a basis for integrated biorefinery of leaves [119]
2015	Papaya (*Carica papaya* L.)	Processing waste	Lycopene	China	Ultrasound extraction (600 W, 40 kHz) Solvents: ethanol/ethyl acetate Extraction time: 15–40 min Temperature: 20–70 °C	Optimization of ultrasound-assisted extraction of lycopene from papaya processing waste by response surface methodology [77]
2015	Carrots, green beans, leeks and celeriac	Vegetable waste streams (rejected carrots, carrot steam peels, green beans cutting waste, leek cutting waste and celeriac steam peels)	Pectin	Belgium	Alcohol insoluble residue Solvents: ethanol and acetone	Pectin characterization in vegetable waste streams: a starting point for waste valorization in the food industry [178]
2015	Berries of *A. melanocarpa*	Black chokeberry wastes	Antioxidants	France	Extraction-adsorption process Extraction time: 2–8 h Temperature: 22 °C	Pilot scale demonstration of integrated extraction-adsorption eco-process for selective recovery of antioxidants from berries wastes [179]
2015	Cashew nuts (CNS)	Shells	Anacardic acid	Tanzania	Review Focus on natural anacardic acids from CNS and other plants and their semi-synthetic derivatives as possible lead compounds in medicine	Potential biological applications of bio-based anacardic acids and their derivatives [180]
2015	Soy, sugarcane, tea	Soy sauce residues, sugarcane bagasse and tea dregs	Hemicelluloses	China	Ionic liquid Solvents: ionic liquids Extraction time: 1–5 h Temperature: 70–100 °C	Quantitative industrial analysis of lignocellulosic composition in typical agro-residues and extraction of inner hemicelluloses with ionic liquid [134]
2015	Tomatoes	Processing tomato	Nutritional bioactive compounds, lycopene	Italy	Biocompatible technology extraction	Recovery of tomato bioactive compounds through a biocompatible and eco-sustainable new technology for the production of enriched “nutraceutical tomato products” [181]
2015	*Citrus sinensis* (Hamlin, Valencia, Pera riu and Pera Natal)	Albedo and flavedo	Flavanone	Brazil	Enzymatic process tannase, pectinase and cellulase Extraction time: 30 h Temperature: 40 °C pH 5	Simultaneous extraction and biotransformation process to obtain high bioactivity phenolic compounds from Brazilian citrus residues [110]
2015	Sunflower	Seeds	Oil- (fatty acids and their antioxidant capacities) and water-soluble phase (proteins, carbohydrates and phenolics)	Slovenia	Subcritical water extraction Extraction time: 5–120 min Temperature: 60–160 °C Pressure: 30 bar	Simultaneous extraction of oil- and water-soluble phase from sunflower seeds with subcritical water [103]
2015	Cereals, root crops, fruits, vegetables, oilseeds, meat, dairy products	Food waste	Nutritionally interesting compounds, chemicals and biofuels	Brazil	Review Sub- and supercritical technologies	Sub- and supercritical fluid technology applied to food waste processing [89]
2015	Agricultural biomass	By-products such as durian peel, mango peel, corn straw, rice bran, corn shell and potato peel	Bio-fuel, water soluble sugars and phenolic compounds	Malaysia and Nigeria	Review Sub-critical water	Sub-critical water as a green solvent for production of valuable materials from agricultural waste biomass: a review of recent work [182]
2015	Sugarcane	Sugarcane waste (rind, leaf and bagasse)	Wax/long-chain aldehydes and* n*-policosanols (nutraceutical compounds) triterpenoids	UK and Brazil	Supercritical CO_2_ (scCO_2_) Extraction time: 4 h Temperature: 50 °C Pressure: 350 bar Flow rate: 40 g/min	Sugarcane waste as a valuable source of lipophilic molecules [183]
2015	Mangoes	Peel	Pectin	Germany and Saudi Arabia	Hot-acid extraction Extraction time: 90 min pH 1.5	The arabinogalactan of dried mango exudate and its co-extraction during pectin recovery from mango peel [184]
2015	Coffee	Spent coffee grounds	Tannin compounds	Malaysia	Alkaline extraction Solvent: NaOH Extraction time: 30–90 min Temperature: 60–100 °C	The influence of extraction parameters on spent coffee grounds as a renewable tannin resource [185]
2014	*Eucalyptus globulus* wood	Trimmings of *Eucalyptus globulus* wood veneers	Phenolic compounds	Spain	Aqueous two-phase extraction PEG 2000 and ammonium sulphate Extraction time: 30–390 min Temperature: 25–65 °C	Aqueous two-phase systems for the extraction of phenolic compounds from eucalyptus (*Eucalyptus globulus*) wood industrial wastes [124]
2014	Pomegranates	By-products after winemaking of pomegranate	(poly)phenolic compounds	Spain, Mexico and Italy	Extraction with MeOH 70% (v/v) and sonication	Assessment of pomegranate wine lees as a valuable source for the recovery of (poly)phenolic compounds [186]
2014	Citrus	Peel, pulp and seeds	Several value-added products, such as essential oils, pectin, enzymes, single cell protein, natural antioxidants, ethanol, organic acids, and prebiotics	Greece and Sweden	Review	Biotransformation of citrus by-products into value added products [187]
2014	Olives	Olive solid waste	Natural dye	Tunisia	Aqueous extraction in closed flasks Solvent: NaOH Extraction time: 15–120 min Temperature: 30–90 °C	Development and optimisation of a non-conventional extraction process of natural dye from olive solid waste using response surface methodology (RSM) [125]
2014	Coffee	Waste coffee grounds	Biodiesel production	United Kingdom	Suspended in fresh heptane room temperature	Effect of the type of bean, processing, and geographical location on the biodiesel produced from waste coffee grounds [188]
2014	Grapevine and hazelnut	Grapevine waste and hazelnut skins	Polyphenols content	Italy and France	UAE and MAE Solvents: ethanol, methanol, acetone, butanone, β-cyclodextrin Extraction time: 5–40 min Temperature: 20–60 °C	Efficient green extraction of polyphenols from post-harvested agro-industry vegetal sources in Piedmont [58]
2014	Bamboo	Raw bamboo culm	Lignin	Malaysia	Review Chemical and steam explosion methods	Extraction and preparation of bamboo fibre-reinforced composites [189]
2014	Spruce	Spruce sawdust	Carboxylic acids	Finland	Alkaline extraction Solvents: Na_2_CO_3_ or Na_2_S.9H_2_O Extraction time: 30 min + 30 min; Temperature: 80 °C up to 160 °C and 210 °C	Production of carboxylic acids from alkaline pretreatment byproduct of softwood [120]
2014	Variety of biomass sources (rapeseed, soybean, palm oil and nonedible feedstocks)	Preferably 2nd–4th generation feedstock (non-edible materials as bagasse, oil waste, microalgae, cyanobacteria and microbes)	Biodiesel	Malaysia	Review Supercritical fluid process and catalytic in situ or reactive extraction process	Integration of reactive extraction with supercritical fluids for process intensification of biodiesel production: prospects and recent advances [90]
2014	Cherries	Cherry seeds	Total phenolic content	Brazil and France	Pressurized fluid extraction (PFE) Solvent: anhydrous ethanol Extraction time: 2–10 min Temperature: 40–80 °C	Isolation by pressurized fluid extraction (PFE) and identification using CPC and HPLC/ESI/MS of phenolic compounds from Brazilian cherry seeds (*Eugenia uniflora* L.) [190]
2014	Corn	Corn stover	Lignin	USA	Protic ionic liquid (PIL) Extraction time: 24 h Temperature: 90 °C	Lignin extraction from biomass with protic ionic liquids [135]
2014	Oranges	Peel	d-limonene	United Kingdom	Microwave-assisted extraction 200 W, closed vessel Solvent: hexane Temperature: 70–110 °C	Microwave-assisted extraction as an important technology for valorising orange waste [59]
2014	Sweet Limes	Peel	Antioxidant phenolics	Pakistan	Enzymatic treatment Incubation time: 30–120 min Temperature: 30–75 °C pH 5 to 8	Optimization of enzyme-assisted revalorization of sweet lime (*Citrus limetta Risso*) peel into phenolic antioxidants [111]
2014	Artichoke	Artichoke scraps	Phenolic compounds	Italy	Ultrasound-assisted extraction (UAE) Time: 60 min Solvent: water	Phenols and antioxidant activity in vitro and in vivo of aqueous extracts obtained by ultrasound-assisted extraction from artichoke by-products [79]
2014	*Cachrys pungens* Jan (Umbelliferae)	Aerial parts of *Cachrys pungens* Jan (Umbelliferae)	Bioactive compounds	Italy	Solvent extraction Solvents: methanol Extraction time: 72 h room temperature dark conditions	Phytotoxic activity of *Cachrys pungens* Jan, a Mediterranean species: separation, identification and quantification of potential allelochemicals [191]
2014	Wheat	Wheat straw	Major organic components (e.g., *N*-heterocycles, fatty acids, phenols and lignins)	Canada	Fast pyrolysis steel shots 475 °C	Wheat straw biomass: a resource for high-value chemicals [192]
2013	Cranberries	Cranberry juice and pomace	Polyphenolics	Canada and Mexico	Pilot scale methods Solvents: ethanol Extraction time: 24 h	Bioactivities of pilot-scale extracted cranberry juice and pomace [48]
2013	Fruits, vegetables, eggs, shrimp	Plant residues, industrial and post-harvest materials	Carotenoids	Mexico	Review Novel environmentally friendly solvents (e.g., ethyl lactate, bioethanol, vegetal oil, commercial enzymes)	Carotenoids extraction and quantification: a review [193]
2013	Tomatoes	Peels	Lycopene	Italy	Enzymatic-assisted extraction Temperature: 45 and 60 °C pH 4–5 and 9–10.5	Environmentally friendly lycopene purification from tomato peel waste: enzymatic-assisted aqueous extraction [112]
2013	Coffee	Coffee residue left after the preparation of the brew (spent coffee grounds—SCG)	Polysaccharides	Portugal	Alkali extraction Solvent: H_2_O and 4 M NaOH Extraction time: 3 h Temperature: 20–120 °C	Extractability and structure of spent coffee ground polysaccharides by roasting pre-treatments [194]
2013	Coffee	Spent coffee grounds	Lipids, oil	Iran	Soxhlet, UAE, MAE, SFE Solvents: petroleum benzene and *n*-hexane Soxhlet: 6 h, boiling temperature UAE: 45 min, ambient conditions MAE: 30 s, 200 and 800 W SFE: 200–250 bar, 40–60 °C, modifier (water, ethanol, hexane)	Extraction of lipids from spent coffee grounds using organic solvents and supercritical carbon dioxide [60]
2013	Forest Industry	Forest residues, including bark	Bioactive molecules	Canada	Review Green alternatives for the design, formulation, and manufacture of new products with applications in various markets (cosmetics, natural health products, biocides, adhesives, coatings)	Forest extractives, the 4th pathway of the forest biorefinery concept [195]
2013	Coffee	Spent coffee grounds (SCG)	Lipid fraction	Portugal and Brazil	Supercritical carbon dioxide Extraction time: 1 h Temperature: 55 °C Pressure: 250 bar CO_2_ flow rate: 15 kg/h	From coffee industry waste materials to skin-friendly products with improved skin fat levels [91]
2013	Walnuts	Green husk	Natural compounds with antioxidant and antimicrobial properties	Spain and Portugal	Solvent extraction Solvents: water, methanol, ethanol Extraction time: 45 min room temperature	Influence of solvent on the antioxidant and antimicrobial properties of walnut (*Juglans regia* L.) green husk extracts [49]
2013	Coffee	Spent coffee	Antioxidants	Spain	Soxhlet, SPE, filter coffeemaker Solvents: water, ethanol, methanol Extraction time: 6–165 min Temperature: 80–100 °C	Influence of extraction process on antioxidant capacity of spent coffee [50]
2013	Tomatoes	Peel	Fatty acids	France	Depolymerization 1.5 M KOMe overnight treatment at room temperature	Interfacial properties of functionalized assemblies of hydroxy-fatty acid salts isolated from fruit tomato peels [196]
2013	Coffee	Spent coffee grounds (SCG)	Polysaccharides	Portugal	Microwave superheated water extraction Extraction time: 5 min Temperature: 200 °C	Microwave superheated water extraction of polysaccharides from spent coffee grounds [61]
2013	Turkish red pine timber	Waste barks	Natural dye	Turkey	Natural dyestuff extraction machine Solvents: water and ethanol Extraction time: 24 h (osmosis)	Natural dye extraction from waste barks of Turkish red pine (*Pinus brutia* Ten.) Timber and eco-friendly natural dyeing of various textile fibers [126]
2013	Cotton, jute, flax, hemp, ramie and natural colorants	Wastes and manufacturing by-products	Fibres, polysaccharides, dyes and pigments, polyphenols, oils and other biologically active compounds	India	Review Conventional maceration, soxhlet, MAE, SFE, ultrasonic extraction	Perspectives for natural product based agents derived from industrial plants in textile applications: a review [197]
2013	Coffee	Spent coffee grounds	Natural antioxidants	Italy	Solvent extraction Solvents: H_2_O, ethanol, Extraction time: 30 min Temperature: 60 °C	Recovery of natural antioxidants from spent coffee grounds [198]
2013	Feijoa fruits	Primarily skin and some flesh	Total soluble solids (TSS), pectin fibre content, total extractable PP content (TEPC) and total antioxidant activity	New Zealand	Accelerated solvent extraction Solvents: (acidified) water, ethanol Temperature: 20 or 50 °C	Utilisation potential of feijoa fruit wastes as ingredients for functional foods [127]
2012	Green tea	Green tea waste	Noncaffeine tea polyphenols	China	Water bath 20 min 90 °C	A novel way of separation and preparation non-caffeine tea polyphenols from green tea waste [199]
2012	Larch	Larch wood-derived lignocellulosic residue	Arabinogalactan, pectin, and crystalline glucose	Russia	Water extraction Extraction time: 2–3 h Temperature: 60–80 °C	An eco-friendly technology for polysaccharide production from logging and sawing waste [128]
2012	Olives	Olive leaves	Oleuropein	Greece	SFE and PLE SFE: 30 MPa, 50 °C, 9.6 kg/h PLE: 10.34 MPa, 10 min, 40–150 °C Solvents: H_2_O and EtOH	Development of a green extraction procedure with super/subcritical fluids to produce extracts enriched in oleuropein from olive leaves [92]
2012	Wood	Wood barks, obtained from pulp mills as industrial wastes	Natural phenolic polymers of tannins and lignin	France	Aqueous extraction urea and sulfite used as water-additives Extraction time: 1 h under reflux Temperature: 75 °C	Development of green adhesives for fibreboard manufacturing, using tannins and lignin from pulp mill residues [129]
2012	Wheat	Wheat milling by-products	High quality oil and vitamin E	Italy	Review Solvent extraction, mechanical pressing or the eco-friendly supercritical carbon dioxide (SC-CO_2_) extraction technology	Durum wheat by-products as natural sources of valuable nutrients [200]
2012	Tree bark	Waste product from paper pulp industries	Antioxidants	Sweden	SFE, PFE, SLE Solvents: scCO_2_, ethanol, H_2_O Extraction time: 30 min–24 h Temperature: 70–180 °C	Extraction of antioxidants from spruce (*Picea abies*) bark using eco-friendly solvents [93]
2012	Timber	Empty fruit bunches	Fiber	Malaysia	Perspective paper	Fiber resin matrix composites: nature’s gift [201]
2012	Oranges	Peel	Essential oil	United Kingdom	Steam distillation and microwave irradiation SD: water, 1 h MW: 12.5 min, 200 °C, power gradient from 400 to 1200 W	*p*-cymenesulphonic acid: an organic acid synthesized from citrus waste [202]
2012	Black tea	Black tea wastes	Pancreatic lipase-inhibiting polyphenols	Japan	Hot-compressed water (HCW) ion-exchange water extraction temperature: 100–200 °C	Polyphenols extracted from black tea (*Camellia sinensis*) residue by hot-compressed water and their inhibitory effect on pancreatic lipase in vitro [203]
2012	Green tea	Green tea waste	Polyphenols	China	Liquid–liquid extraction Solvents: H_2_O, glyceryl, triacetate, *n*-butanol, ethyl acetate Extraction time: 12 h + 2 h	Recovery of tea polyphenols from green tea waste by liquid–liquid extraction [204]
2012	Citrus	Peels	Polymethoxy flavonoids	China	Solvent extraction Solvents: methanol and ethanol Extraction time: 1–3 h Temperature: 65–85 °C	Study on the extraction technique of poly-methoxyflavonoids from citrus peels by using response surface methodology [205]
2011	Coffee	Husks	Caffeine	Spain	Supercritical CO_2_ Extraction time: 20 min Temperature: 323 K Pressure: 60 bar CO_2_ flow rate: 2–3 g/min	Extraction of caffeine from Robusta coffee (*Coffea canephora* var. Robusta) husks using supercritical carbon dioxide [94]
2011	Oranges	Peel	Essential oils	France and Tunisia	Microwave steam diffusion (MSDf) Extraction time: 12 min Temperature:100 °C	Microwave steam diffusion for extraction of essential oil from orange peel: kinetic data, extract’s global yield and mechanism [62]
2011	Grape	Skins	Anthocyanins	Spain	Microwave-assisted extraction Solvents: H_2_O, methanol Extraction time: 5–20 min Temperature: 50–100 °C	Microwave-assisted extraction of anthocyanins from grape skins [63]
2011	Tea (green, oolong and black)	Tea residues (green, oolong and black tea residues)	Phenolic compounds	Japan	Microwave-assisted extraction water under autohydrolytic conditions Extraction time: 2 min Temperature: 110–230 °C	Microwave-assisted extraction of phenolic compounds from tea residues under autohydrolytic conditions [64]
2011	Sea Buckthorn (Hippophae rhamnoides	By-Products of juice production	Flavonoids	France	Solvent-free microwave hydrodiffusion and gravity (MHG) without addition of solvent or water atmospheric pressure	Solvent free microwave-assisted extraction of antioxidants from sea buckthorn (*Hippophae rhamnoides*) food by-products [206]
2011	Wheat	Wheat straw	Energy and CO_2_ secondary metabolites including fatty acids, wax esters and fatty alcohols	England	Supercritical CO_2_ extraction Temperature: 40–100 °C Pressure: 100–300 bar CO_2_ flow rate: 40 g/min	Use of green chemical technologies in an integrated biorefinery [95]
2011	Olives	By-products generated during storage of extra virgin olive oil	Phenolic compounds, hydroxytyrosol, tyrosol, decarboxymethyl oleuropein aglycone, and luteolin	Italy and Spain	Solid–liquid and liquid–liquid extraction Solvents: n-hexane, methanol, H_2_O Extraction time: 1 h	Wastes generated during the storage of extra virgin olive oil as a natural source of phenolic compounds [207]
2010	Tomatoes	Ground tomatoes without seeds	Lycopene	France and Algeria	Solvent extraction Solvent: d-limonene	Carotenoid extraction from tomato using a green solvent resulting from orange processing waste [208]
2010	Tea plant	Tea stalk and fiber wastes	Caffeine	Turkey	Supercritical CO_2_ ethanol as co-solvent Extraction time: 1–5 h Temperature: 50–70 °C Pressure: 250 bar semi-continuous flow	Effect of ethanol content on supercritical carbon dioxide extraction of caffeine from tea stalk and fiber wastes [96]
2010	Portuguese elderberry	Pomace	Anthocyanins	Portugal	Supercritical CO_2_ extraction Solvents: CO_2_, water, ethanol Extraction time: 40 min Temperature: 313 K	Effect of solvent (CO_2_/ethanol/H_2_O) on the fractionated enhanced solvent extraction of anthocyanins from elderberry pomace [97]
2010	Green tea	Green tea waste	Polyphenols, total catechins, and reducing sugars	South Korea and USA	Solvents: cold water (25 °C), hot water (90 °C), sulfuric acid, hydrochloric acid and methanol Extraction time: 20 min 250 rpm	Effects of cellulase from *Aspergillus niger* and solvent pretreatments on the extractability of organic green tea waste [130]
2010	Tea	Tea waste	Caffeine	Iran	Subcritical water extraction Temperature: 100–200 °C Pressure: 20–40 bar water flow rate: 1–4 g/min	Isolation of caffeine from tea waste using subcritical water extraction [104]
2010	*Citrus sudachi*	Peels	Flavones	Japan	Microwave-assisted extraction Solvents: methanol extraction time: 10 to 12 min	Microwave-assisted extraction and methylation of useful flavones from waste peels of *Citrus sudachi* [209]
2010	Mate (*Ilex paraguariensis*)	Mate residue	Compounds with antioxidant properties, such as phenolic acids and methylxanthines, such as caffeine	Brazil	Solvent extraction Solvent: methanol, H_2_O, ethanol sonication for 15 min room temperature	Phenolic acids and methylxanthines composition and antioxidant properties of mate (*Ilex paraguariensis*) residue [210]
2010	Rice	Rice bran	Phenolic compounds as well as other valuable materials	Japan	Subcritical water Preheated oil: 100–180 °C, 10 min Preheated water bath: 180–360 °C, 10 min and 220 °C for 2–30 min	Production of phenolic compounds from rice bran biomass under subcritical water conditions [105]
2009	*Citrus*	Peels	Essential oil	France and Algeria	Microwave hydrodiffusion gravity Extraction time: 15 min atmospheric pressure 500 W	A new process for extraction of essential oil from citrus peels: microwave hydrodiffusion and gravity [65]
2009	Kiwifruit	By-products derived from kiwifruit processing	Phenolics and pectin polysaccharides	New Zealand	Solvent extraction Solvents: water, ethanol Extraction time: 1 h room temperature	Evaluation of the extraction efficiency for polyphenol extracts from by-products of green kiwifruit juicing [211]
2009	Palm	Black liquor of oil palm waste	Lignin	Malaysia	Solvent extraction Chemical extractions: di-ethyl ether, alcohol-benzene mixture treatment with H_2_SO_4_ for 30–45 min	Exploring the antioxidant potential of lignin isolated from black liquor of oil palm waste [212]
2009	Turkish tea plants	Tea stalk and fiber wastes	Caffeine	Turkey	Supercritical carbon dioxide Extraction time: 1–10 h Temperature: 55–75 °C increasing pressure up to 250 bar semi-continuous flow	Extraction of caffeine from tea stalk and fiber wastes using supercritical carbon dioxide [99]
2009	Rice	Rice bran	Oil (value-added materials such as amino acids, organic acids, and water-soluble saccharides)	Japan	Subcritical water preheated oil bath: 100–180 °C Preheated salt bath: 200–360 °C Reaction time: 5 min	Sub-critical water treatment of rice bran to produce valuable materials [106]
2009	Several biomass	Residues rich in lignocellulosics	Bio-based chemicals (e.g., succinic, lactic, fumaric l-malic, l-aspartic acids)	England	Review Focus on green chemical conversion of lignin into higher value chemicals	The integration of green chemistry into future biorefineries [21]
2009	Apple	Industrially generated apple pomace	Antioxidants and polyphenols	Ireland	Pressurized liquid extraction accelerated solvent extractor static extraction of 5 min Temperature: 75–193 °C	The optimization of extraction of antioxidants from apple pomace by pressurized liquids [213]
2008	Chicory, citrus, cauliflower, endive, and sugar beet	Plant by-products (chicory roots, citrus peel, cauliflower florets and leaves, endive, and sugar beet pulps)	Pectins	France and Finland	Enzymatic extraction Extraction time: 4 h Temperature: 50 °C	Extraction of green labeled pectins and pectic oligosaccharides from plant by-products [113]
2008	Tea (green, oolong, and black)	Green, oolong, and black tea residues	Polysaccharides, polyphenols, arabinose, galactose, xylose, catechins	Japan	Microwave heating Solvent: water Temperature: 110–230 °C	Microwave heating of tea residue yields polysaccharides, polyphenols, and plant biopolyester [66]
2008	Plant lipids	Plant oils and other natural lipidic phases	Phytosterols, vitamins	Czech Republic	Review Enzymes as efficient natural catalysts	Plant products for pharmacology: application of enzymes in their transformations [114]
2007	Broccoli	Broccoli seeds	Natural sulforaphane	China and Australia	Liquid–liquid and solid-phase extraction Solvents: ethanol, hexane, ethyl acetate	Separation and purification of sulforaphane from broccoli seeds by solid phase extraction and preparative high-performance liquid chromatography [214]
2006	Tea	Tea waste	Caffeine	Turkey	Solid–liquid extraction solvents: hot water and chloroform Temperature: 370 K and 293 K	Solid–liquid extraction of caffeine from tea waste using battery type extractor: process optimization [215]

**Fig. 5:**
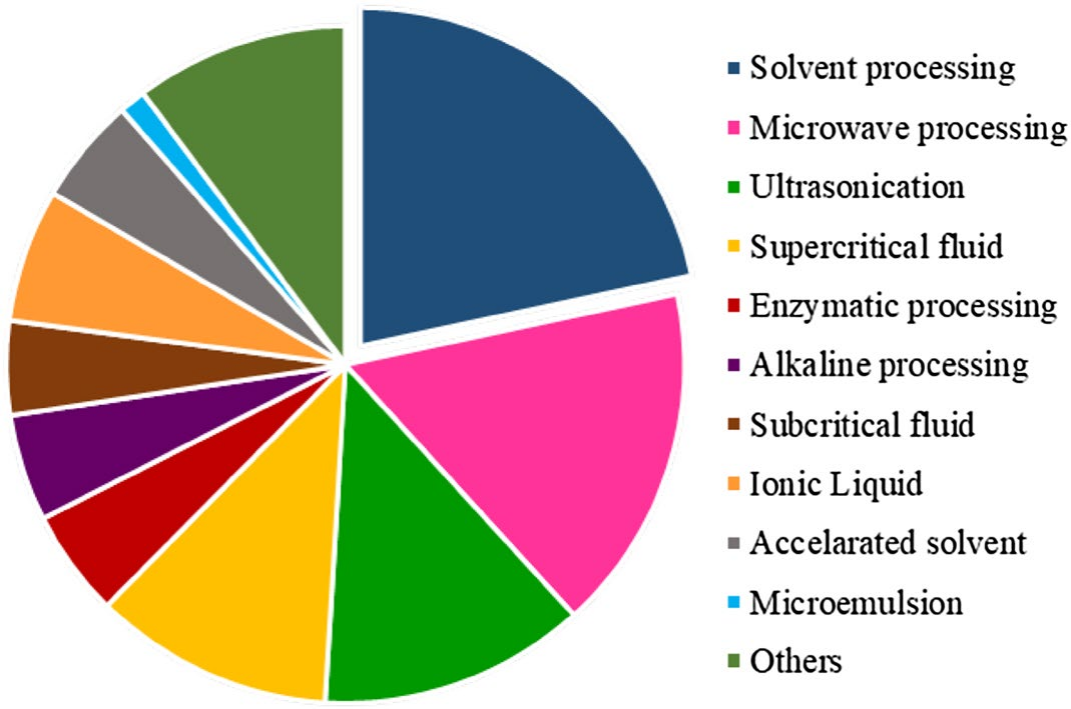
Main green and sustainable techniques used to separate natural products from waste described in research papers (ISIS Web of Knowledge, January 2006 to December 2017)

The decision concerning the best method to separate the compounds of interest from the raw material is dependent on several aspects, such as the characteristics of the target extracts and raw material (physical–chemical properties), available technology, required purity, selectivity, stability and, more importantly here, the greenness of the whole process. As can be seen in Fig. 5, the most cited techniques in these research papers were based on solvent/maceration (25% of the total), microwave (19%), ultrasonication (14.7%) and supercritical fluid processing (13%), followed by methods using ionic liquids (7%), enzymatic and subcritical fluid treatment (6%), as well as the association of two or more techniques.

According to the literature, the most widespread approaches for separating natural products from a number of matrices are based on liquid–liquid or solid–liquid extraction (LLE and SLE). Several greener alternatives have been proposed by replacing toxic or non-renewable organic solvents, as well as the extraction times. In some cases, solid-phase extractions (SPE) were also carried out and decreased both the amount of solvent and the number of extraction cycles, offering high enrichment factors [39, 40]. Actually, the mass transfer enhancement for SLE has been largely studied and applied, contributing to technology innovation, process intensification and integration, and energy saving, especially important for microwave, ultrasound, and high-pressure processing, for instance [41]. An overview of these techniques and related examples will be discussed in this section.

### From Conventional Solvent Separation to Enhancement Processing Approaches Over the Last 10 Years

Solvent processing is one of the most traditional methods to remove natural products from bio-derived materials. In this extraction approach, the raw material in adequate size is exposed to different solvents, mostly organic, which remove soluble components of interest. The samples are then usually centrifuged and filtered to separate the solid residue, and the extract is used in this way (as a food supplement or for preparing functional foods, for example) or treated after this step. Solvent extraction is attractive compared to other methods due to low cost and simplicity. However, this method does not always use benign solvents; it frequently requires an evaporation/concentration step for recovery, it usually demands large amounts of solvent and needs a long time to be carried out. Additionally, the possibility of thermal degradation of natural bioactive components is also possible due to the high temperatures used during the extraction process [42]. Despite this, it is largely used in industries, where solvent reuse is of great economic importance. In general, the raw material (in its liquid or solid form) is mixed with a solvent, and the separation kinetic of the target compounds is influenced by parameters such as the solvent ratio, pH, and temperature and, for SLE, the particle size. The solvent should be atoxic, non-flammable and stable at working conditions, ideally renewable and cheap, with low viscosity and an adequate boiling point, allowing for easier solvent removal from the extract/fraction [43]. Recently, several models have been proposed to predict the best solvents to be used in a specific case, which do not only take into account physical descriptors, such as enthalpy of vaporization, dielectric constant, refractive index, boiling point, etc., but also empirical descriptors to evaluate, for instance, intermolecular forces (specific and non-specific solute–solvent interactions, e.g., hydrogen bond donor and/or hydrogen bond acceptor, Van der Waals and ion/dipole forces). Purely theoretical descriptors have been also introduced, offering the most important advantage of not requiring any experiments, as is the case of the model known as quantitative structure property relationship (QSPR), able to predict 127 polarity scales for more than 700 solvents [44].

The solvent selection also depends on the physical–chemical proprieties of the compounds of interest, considering principally the selectivity and greenness degree of the process, aiming at obtaining high recoveries and the integrity of the target compounds. In general, the raw material stays in contact with the solvent for a certain period (from minutes to days), when the soluble compounds are transferred from the matrix to the extractor phase, usually by shaking the system. For SLE, the dispersion of the particles in the solvent is facilitated agitating them, optimizing their contact and accelerating the separation process. Traditionally, solvent treatment is performed at room temperature, although heating can promote higher recoveries to these compounds that are not thermosensitive. In some cases, LLE and SLE can be time-consuming, demanding further purification and concentration steps, which are their main drawbacks [41, 45].

Maceration using green and non-toxic solvents for the separation of natural products from plant-derived waste has been described over the last years (e.g., to remove dyes from quince leaves or catechins, theaflavins, gallic acid, and antioxidants in general from walnut green husk, cranberry pomace, black tea and banana processing waste). According to these studies, using water, methanol, ethanol or a mixture of them at 70–100 °C can be a low-cost, benign alternative for the recovery of high added-value compounds derived from residual biomass [46–49]. Scaling-up was also studied, whose results showed to be useful in determining industrial process feasibility and the economic value of polyphenols for commercial use, increasing the overall profitability of the cranberry industry [48].

Whenever possible, higher temperatures allow for higher mass transfer in a shorter time with lower energy consumption in general, resulting in better recovery efficiency than conventional systems [50]. As observed in Fig. 5, the second most cited green and sustainable separation process is based on microwave heating and can be considered a non-conventional technique nowadays. Heating is based on non-ionizing electromagnetic waves. Those between 0.915 and 2.45 GHz are used for industrial, scientific and medical applications. The overall principle of heating is rooted in its direct impact with polar materials/solvents and is dependent on ionic conduction and dipole rotation, occurring simultaneously in most cases. The increased temperature can overcome the natural product-matrix interaction caused by Van der Waals forces, dipole attraction, hydrogen bonding of the compounds of interest and active sites in the matrix. Therefore, thermal energy can disrupt both solute–solute and solute–matrix interactions, providing the activation energy required for the desorption process. The mass transfer of the compounds from the raw material to the solvent is also accomplished by convection and diffusion mechanisms, causing the explosion of plant cells and releasing their content into the liquid phase [51].

The eco-friendly removal of essential oils, pectin and polyphenols from a number of plant raw materials mediated by microwave irradiation has been described over the last years, paying special attention to citrus waste [52–66]. In fact, the orange juice processing industry can be considered more than a good case study. This sector is highly wasteful, generating 50% of waste from the total fruit/starting material (e.g., peel, bagasse, seeds and yellow water). Around 20 million tonnes of orange peel per year are produced worldwide, which consist of water (80%) and sugars, cellulose, hemicellulose, pectin and d-limonene (20%). Recently, it was shown using a mathematical model that d-limonene extraction consisted of a two stage diffusion process for a microwave (MW) heating approach: initial extraction from the exterior of cells followed by trans-membrane diffusion. Compared to other conventional extraction methods, it was found that the microwave treatment was more efficient, resulting in a higher overall yield due to the access to a higher amount of d-limonene [59].

The successful microwave-assisted solvent-free modification of pectin derived from citrus waste has also been reported [53]. These approaches not only allow for the separation of the major components of citrus peel, but they also add further value through the production of other high value-added products, such as pectin, d-limonene and a rare form of mesoporous cellulose which are produced in a single step, without added acid [67]. Along these lines, the concept of dry-biorefinery is gaining momentum, since valuable products can be recovered from plant by-products without adding solvents or water, using green processes such as MW [56]. Innovation relies on the separation of the target compounds from raw materials, which are rich in water, achieved without adding solvents or water, illustrating a circular systemic process; i.e., all materials and resources could be reintegrated into the integrated and zero-waste biorefinery [19]. Although very attractive, as expected, the design and use of real MW industrial scale equipment requires additional studies related to safety, corrosion and maintenance intervals [68].

The combination of two or more extraction/concentration methods is quite common in the literature (Table 1). As described by Boukroufa et al. [56], the removal of essential oil, polyphenols and pectin from orange waste was conducted using microwave and ultrasound technology, without adding any solvents. Essential oil separation was performed by Microwave Hydrodiffusion and Gravity (MHG), and thereafter the remaining water of this process was used as a solvent for the subsequent extraction of flavonoids and pectin. For polyphenol separation, ultrasound-assisted extraction (UAE) was used, and response surface methodology (RSM) using the central composite design (CCD) approach was used to investigate the influence of some variables. The CCD revealed that the optimized conditions of ultrasound power and temperature were 0.956 W/cm^2^ and 59.83 °C giving a polyphenol yield of 50.02 mg GA/100 g dm, which, compared to conventional extraction, promoted an increase of 30% in the yield. Pectin was extracted by microwave-assisted extraction, resulting in a maximal yield of 24.2% for microwave power of 500 W (3 min), whereas traditional extraction provides18.32% (120 min). As can be seen, the combination of microwave, ultrasound and recycled water resulted in higher recoveries of the compounds of interest in a shorter time, so that a systemic loop/cycle could be closed using only the resources generated in the plant. This makes the whole process optimized in terms of time, energy savings, cleanliness and reduced amount of waste.

As can be noted, ultrasound has been widely utilized for helping to extract target components from waste plant-derived sources, reducing separation time, solvents, energy consumption and improving the product quality. The effectiveness of ultrasound is attributed to the cavitation phenomenon, assisting the solubilization of the compounds of interest into the solvent, enhancing their removal from the bulk raw material [69]. According to Chemat [70], the ultrasound waves (from 20 kHz to 10 MHz) pass through an elastic medium, inducing a longitudinal displacement of particles resulting in a succession of compression and rarefaction phases in this medium. Every medium has a critical molecular distance and, below this critical point, the liquid remains intact. However, above this distance, the liquid would break down, creating voids (cavitation bubbles) in the liquid. When the size of these bubbles reaches a critical point they collapse, releasing a large amount of energy. The estimated temperature and pressure at this time are estimated at 5000 and 2000 K atmospheres. This creates hotspots that accelerate the chemical reactivity into the medium, generating microjets directed towards the solid surface, also responsible for the general higher effectiveness of this technique, as the high pressure and temperature involved in the process destroy the cell walls of the plant matrices and their content can be released into the medium more easily.

Some new process aiming at agro-industrial waste application in food industries based on ultrasound-assisted extraction of natural products have been reported [71–79], as is the case of carotenoid separation from pomegranate peels using different vegetable oils as solvents [72]. Sunflower and soybean oils were used as solvents and parameters such as time, temperature, solid/oil ratio used were analyzed considering the yield. It was found that the optimum mild operating conditions were: extraction temperature, 51.5 °C; peel/solvent ratio, 0.10; amplitude level, 58.8%; solvent, sunflower oil. Additionally, a subsequent separation of oil and carotenoids was not necessary, since the pigmented oil can be used as a carotenoid source in different commercial products in this format.

The green recovery of cellulose from oil palm bunches by autoclave-based and ultrasonication pre-treatments were successfully developed to replace the non-green chlorite method [73]. An ultrasonic process with hydrogen peroxide yielded 49% cellulose with 9.13% alpha-cellulose content and 68.7% crystallinity, as compared to 64% cellulose with an autoclave treatment. The cellulose/polypropylene composites generated with high tensile strength, high thermal stability, and low water and diesel sorption showed great potentials for conversion into eco-composite products such as polymeric material insulated cables for high voltage engineering, automotive parts, sports tools and other household or office items.

Another highly cited green and sustainable technique to isolate organic compounds from bio-based waste is based on supercritical fluid processing (Fig. 5). It is widely known that substances at temperatures and pressures near or above their critical points have exceptional solvent characteristics for analytical purposes. These supercritical fluids possess liquid-like solvating and gas-like diffusivity power, and other tuneable properties that can be adjusted varying temperature, pressure and the addition of other components acting as a modifier. Due to its gas-like low viscosity and high diffusivity, the supercritical fluid can easily penetrate into plant materials with a fast mass transfer rate. Possibly, the most important property of supercritical fluids for separation processes is diffusion, obtaining solubility and diffusion good enough to provide quantitative extraction yield [80, 81]. Carbon dioxide (scCO_2_) is the fluid most widely used for extractions, with critical parameters of 31.1 °C and 73 atm (7.39 MPa), at relatively low operating conditions. It behaves as a nonpolar or polarizable solvent and low molar mass alcohols (co-solvents) are often added in small quantities to modify the solvent polarity. Because carbon dioxide can be depressurized to the gaseous state, the solvent is easily removed and supercritical fluid-based separation methods are easily coupled with subsequent analysis. Therefore, scCO_2_ provides miscibility to the majority of natural products, availability and low cost, reliably high purity, negligible toxicity, facility for removal and reuse, resulting in many advantages for downstream processing in terms of product purification and/or catalyst recycling [80].

The approach using scCO_2_ has been widely used for isolation and purification of chlorophylls, carotenoids, lipids, alkaloids, antioxidants from matrices such as filter tea, spruce bark, tomato and elderberry pomace, grape, passiflora, coffee and cupuassu seed waste [82–99]. In addition to the optimization of the separation process, some studies also aim to evaluate the techno-economic viability of large-scale commercial production, for example, to obtain cupuassu butter from cold-pressed seed residues, also evaluating the influence of thermodynamic and kinetic variables of yield, chemical composition and production costs of the extracts [86]. Optimal conditions related to extraction kinetics, chemical composition and production costs were 30–35 MPa and 50 °C. It was shown that the phenolic content (0.47–2.82 mg/g) was lower than those commonly found using other methods (20–23 mg/g). The high contents of tocopherols, as well as the unsaturated fatty acids (48%) compared to the saturated fatty acids (52%) present in the butter obtained by scCO_2_ demonstrated its great potential as an ingredient in food, pharmaceutical and cosmetic industries. In addition, process intensification for biodiesel production involving supercritical fluids has been reported [84, 90]. Such approaches can allow biodiesel production without any addition of catalyst, or via catalytic in situ or reactive extraction process, combining the extraction and reaction phase together in a single operation unit. These studies also discuss both processes towards the future bio-refinery setup and more efficient use of all waste produced.

The use of fluids different to CO_2_ has been described in the literature, but as they are usually organic solvents, they do not show any distinct advantages and often have high critical temperatures. Despite having a very high critical temperature, water shows unique properties in the subcritical region (200–300 °C), as a reduction in dielectric constant (20–30) and density (0.7–0.8 g/cm^3^) compared to water at room temperature, improving its ability to dissolve nonpolar organic and inorganic compounds. Under these conditions, the water dissociation constant into hydroxide and hydrogen ions are more than three orders of magnitude higher, so that near-critical water acts as a self-neutralizing acid or base catalyst, avoiding salt waste generation. Moreover, using subcritical and supercritical water conditions greatly simplifies the product purification step in some cases, since nonpolar products are insoluble in water in lower temperatures [80, 100–106].

Other potential scalable approaches have been described, such as enzymatic [107–114], alkaline [115–120] and based on different types of aqueous media (e.g., cyclodextrins, montmorillonite K-10/LiOH, green liquor) [121–130]; ionic liquids [131–135], deep eutectic solvents [136–138], constituting alternative methods for the recovery of high added-value compounds from agro-industrial waste aiming at obtaining the best analytical, economical and socio-environmental compromise [139–142].

Based on the investigated literature [143], Table 2 summarizes the advantages and disadvantages of the four most cited green and sustainable techniques.

**Table 2:** Advantages and disadvantages of different technologies that were most cited as green and sustainable techniques over the last 10 years

	Advantages	Disadvantages
Solvent processing	Inexpensive and simplicity; allows for solvent reuse	Does not always uses benign solvents; frequently requires an evaporation/concentration step for recovery; usually demands large amounts of solvent and long extraction time; possibility of thermal degradation
Microwave processing	Reduced extraction time; reduced solvent usage; improved extraction yield; simple and inexpensive	Not good when either target compounds or solvents are non-polar or volatiles
Ultrasonication	Inexpensive, simple and efficient; can reduce the operating temperature (good for thermolabile compounds); can be used with any solvent	Its efficiency may be linked to the nature of plant matrix; the active part of ultrasound inside the extractor is restricted to a zone located in the vicinity of the ultrasonic emitter
Supercritical fluid	Moderate extraction temperature (good for thermolabile compounds); rapid mass transfer (larger extraction rate); solubility of a chemical in a supercritical fluid can be manipulated; can eliminate concentration process; the solutes can be separated from supercritical fluids without losing volatiles due to its extreme volatility; additional filtration or centrifugation to remove solid residue is not necessary	Onerous operating conditions

## Conclusions

The establishment of vanguard biorefineries for bioeconomy and circular economy urgently demands innovation in green and sustainable separation for the recovery of natural products from agro-industrial by-products all over the world. Sustainable separation includes the idea of integrated valorization not only in an economic sense, but also strengthens other social and environmental dimensions, from small to large producing scales. According to the literature over the last decade, the number of studies in this field has grown significantly in recent years. New approaches incorporating holistic extraction and/or purification techniques, also integrating systemic chemical transformation through the design and use of renewable materials and optimized processes should combine the best green analytical figures of merit with online evaluation of the whole production chain. These approaches should generate healthier and more efficient products, methods and processes at an affordable and fair cost.

Overall, solvent processing and its modification towards the enhancement of mass transfer to remove the compounds of interest from selected waste have been widely used (25%), also on industrial scales. Alternative extraction or purification methods have shown increasingly more applications, such as for microwave, ultrasonication and supercritical fluid processing. It was shown that a wide range of natural products and their derivatives are used mainly in food (as dyes, aromas, flavors) in medicines or green formulations in agriculture. According to the data available, one paradigmatic case largely studied is the valorization of citrus waste, representing more than 10% of all residues considered in the research papers.

Moreover, an emergent challenging topic is to evaluate biorefinery processing alternatives, i.e., sustainability assessment tools, for example LCA, which include parameters such as feedstock supply (to verify the suitability and adequacy of a potential biomass feedstock for the separation or transformation treatment), process performance (to assess the input–output balance of material and energy flows) and bio-based chemical production [144]. Therefore, the decision about the best separation approach takes into account various fundamental aspects and is based on green and sustainable assessment tools, considering the type of agro-industrial waste (e.g., quantity, periodicity, chemical variability, water amount, distance to the processing unit), the natural target products (chemical quality, purity, humidity, costs etc.) and available technologies.

Using sustainability indicators and tools will be increasingly demanded in this field, contributing to the greenness or sustainability of the whole processing system. The development of a sustainable separation method which provides better recovery efficiency will not only add value to the agro-industrial waste, reducing the overall manufacturing costs and the use of synthetic chemicals, but will also aggregate value to the whole production chain, including its final products. The emergence of bio-based industries is changing the current status of the producing systems, contributing to the current biomass residual losses. Based on the literature, the scenario for future research and innovation in green and sustainable separation for the recovery of agro-industrial waste is truly beginning, bringing together various areas and sectors towards more efficient and circular systems.
